# Comprehensive Phytochemical Characterization of *Echinophora tenuifolia* subsp. *sibthorpiana* Reveals Radical Scavenging, Antimicrobial, and Antiproliferative Activities Associated with Apoptosis and G2/M Cell Cycle Accumulation

**DOI:** 10.3390/ijms27167385

**Published:** 2026-08-18

**Authors:** Yılmaz Uğur, Turgay Kolaç, Muhammed Dündar, Rukiye Zengin, Tahir Macit, Umay Sena Alan, Hüseyin Karcı, Zeynep Maraş, Feyza Yılmaz Dündar, Onural Özhan, Selim Erdoğan

**Affiliations:** 1 Health Services Vocational School, Inonu University, Malatya 44280, Türkiye; turgay.kolac@inonu.edu.tr; 2 Department of Molecular Biology and Genetics, Faculty of Arts and Sciences, Inonu University, Malatya 44280, Türkiye; muhammed.dundar@inonu.edu.tr (M.D.); huseyin.karci@inonu.edu.tr (H.K.); yilmazfeyza56@gmail.com (F.Y.D.); 3 Republic of Türkiye Ministry of Agriculture and Forestry, General Directorate of Agricultural Research and Policies, Apricot Research Institute, Malatya 44090, Türkiye; rukiyezengin12@gmail.com (R.Z.); tahirmacit444@hotmail.com (T.M.); 4 Basic Pharmaceutical Sciences Department, Faculty of Pharmacy, Inonu University, Malatya 44280, Türkiye; umaysenalann@gmail.com (U.S.A.); zeynep.maras@inonu.edu.tr (Z.M.); selim.erdogan@inonu.edu.tr (S.E.); 5 Department of Medical Pharmacology, Faculty of Medicine, Inonu University, Malatya 44280, Türkiye; onural.ozhan@inonu.edu.tr

**Keywords:** *Echinophora tenuifolia* subsp. *sibthorpiana*, phytochemical profiling, radical scavenging, antimicrobial activity, antiproliferative activity, apoptosis, cell cycle arrest, LC–MS/MS

## Abstract

*Echinophora tenuifolia* subsp. *sibthorpiana* is an aromatic and traditionally used plant whose phytochemical diversity and pharmacological potential remain incompletely characterized. This study aimed to integrate comprehensive chemical profiling with antioxidant, antimicrobial, and antiproliferative evaluations of an acidified methanolic extract prepared from its aerial parts. Phenolic constituents, volatile compounds, and fatty acids were characterized using LC–MS/MS, GC–MS, and GC–FID, respectively. Antioxidant capacity was assessed using DPPH and ABTS assays, antimicrobial effects were evaluated by disc diffusion and broth microdilution methods, and antiproliferative activity was investigated in six human cancer cell lines and non-cancerous BEAS-2B cells. Apoptosis induction and cell-cycle alterations were further examined by flow cytometry. The extract contained a high total phenolic content of 181.78 ± 5.03 mg GAE/g and exhibited substantial DPPH and ABTS radical scavenging activities of 333.65 ± 2.94 and 262.18 ± 6.80 mg TE/g, respectively. LC–MS/MS analysis identified rutin hydrate, quinic acid, chlorogenic acid, narcissin, and vicenin-2 as the predominant non-volatile constituents. The essential oil was dominated by methyl eugenol (51.10%) and Δ-3-carene (32.23%), whereas lauric acid (29.71%) was the major fatty acid. The extract also exhibited measurable antimicrobial activity and concentration-dependent cytotoxicity, with the greatest sensitivity observed in MCF-7 breast cancer cells (IC_50_ = 32.96 ± 4.80 μg/mL), followed by A549 and MDA-MB-231 cells. However, the relatively close IC_50_ values observed in cancer and non-cancerous BEAS-2B cells indicated limited cancer-cell selectivity under the tested conditions. Flow-cytometric analyses demonstrated marked apoptosis induction in MCF-7 cells, reaching 57.93 ± 1.80%, together with a cell-line-dependent accumulation in the G2/M phase. Collectively, these findings indicate that *E. tenuifolia* subsp. *sibthorpiana* is a chemically diverse source of plant-derived bioactive compounds with notable radical scavenging and measurable antimicrobial and cytotoxic effects. Further bioactivity-guided fractionation and mechanistic studies are required to identify the active constituents and clarify their pharmacological relevance and selectivity.

## 1. Introduction

Medicinal and aromatic plants are important sources of food additives, traditional remedies, and bioactive compounds. Their relevance has increased considerably in recent years due to the growing interest in natural products as alternatives to synthetic agents, particularly in response to adverse side effects, antimicrobial resistance, and the demand for multifunctional compounds with pharmaceutical, cosmetic, and food-related applications [1,2]. Plant-derived secondary metabolites, including phenolic acids, flavonoids, terpenoids, and related constituents, exhibit a wide range of biological activities such as antioxidant, antimicrobial, anti-inflammatory, and cytotoxic effects [3,4,5,6,7,8]. Recent reviews have also highlighted the anticancer potential of food and medicine homology substances, emphasizing their multi-target actions and potential relevance in tumor prevention and treatment [9].

The genus *Echinophora* (Apiaceae) comprises approximately ten species distributed mainly in the Mediterranean basin and the Middle East [3,10,11]. Türkiye represents an important center of diversity for this genus, where six species occur, three of which are endemic [1,4,12]. Members of this genus, commonly known as “Çörtük,” “Tarhana otu,” or “Yıldız Çördüğü,” are widely used as flavoring agents in traditional foods such as tarhana, pickles, and meat products, and have long been employed in folk medicine for the treatment of digestive disorders, gastric ulcers, wound healing, pulmonary complaints, and as diuretic agents [3,4,5,6,10,13].

Among these taxa, *Echinophora tenuifolia* subsp. *sibthorpiana* has attracted attention because of its dual importance as an aromatic food plant and a potential medicinal resource [3,14,15]. Previous studies on this subspecies have mainly focused on its essential oil composition and selected biological activities [11,13]. The volatile fraction has been reported to be dominated by methyleugenol and *α*-phellandrene, which contribute substantially to the characteristic aroma and antimicrobial properties of the plant [13,16,17]. In addition, its antioxidant capacity has been evaluated using DPPH and ABTS assays, and a positive relationship between phenolic content and antioxidant activity has been suggested [7,8]. Several in vitro studies have also demonstrated antibacterial and antifungal activities of *Echinophora* extracts and essential oils against pathogens such as *Staphylococcus aureus*, *Escherichia coli*, and *Candida albicans* [3,4,18].

Despite these findings, the available literature on *E. tenuifolia* subsp. *sibthorpiana* remains limited and fragmented. Although broader pharmacological and cytotoxic investigations have been reported for related taxa such as *E. platyloba*, comprehensive information regarding the biological activities of *E. tenuifolia* subsp. *sibthorpiana* is still scarce [4,19,20,21]. In particular, although antiproliferative activity has been demonstrated in several studies, the underlying mechanisms responsible for cancer cell growth inhibition remain largely unexplored. Since apoptosis induction and cell cycle arrest represent two of the principal mechanisms through which plant-derived phytochemicals suppress tumor progression, investigating these processes may provide valuable insight into the anticancer potential of *E. tenuifolia* subsp. *sibthorpiana*. In addition, antimicrobial studies on *E. tenuifolia* subsp. *sibthorpiana* have largely focused on screening approaches, while quantitative assessment of antimicrobial activity remains limited. Likewise, evaluation across multiple cancer cell lines, including neuroblastoma (SH-SY5Y), breast cancer (MDA-MB-231, MCF-7), lung cancer (A549), colorectal cancer (HCT116), cervical cancer (HeLa), and non-cancerous lung epithelial cells (BEAS-2B), together with mechanistic analyses, may provide a more comprehensive understanding of the biological selectivity and therapeutic relevance of this subspecies [4,19,22,23,24,25].

Therefore, the present study aimed to provide an integrated phytochemical and biological characterization of *E. tenuifolia* subsp. *sibthorpiana*. The phenolic profile, volatile composition, and fatty acid content were analyzed using LC–MS/MS, GC–MS, and GC–FID, respectively. In parallel, the biological activity spectrum of the extract was evaluated through antioxidant assays, antimicrobial screening, determination of minimum inhibitory concentration (MIC), cytotoxic analysis across a panel of healthy and cancerous cell lines, and mechanistic investigations involving apoptosis and cell cycle analyses. By integrating phytochemical profiling with quantitative antimicrobial evaluation and mechanistic anticancer investigations, this study sought to expand the current understanding of this taxon and clarify its potential as a source of multifunctional natural bioactive compounds.

## 2. Results

### 2.1. Total Phenolic Content and Antioxidant Capacity

The TPC and radical scavenging activity of the extract are presented in Table 1. The extract exhibited a total phenolic content of 181.78 ± 5.03 mg GAE/g dry extract. The DPPH and ABTS radical scavenging activities of the extract, determined by DPPH and ABTS radical scavenging assays, was 333.65 ± 2.94 mg TE/g and 262.18 ± 6.80 mg TE/g, respectively.

### 2.2. LC–MS/MS Profile of Phytochemical Compounds

The phytochemical composition of the extract determined by LC–MS/MS analysis is presented in Table 2. The corresponding retention times, MRM transitions, and analytical validation parameters are summarized in Table 3. Among the analyzed compounds, rutin hydrate was the predominant constituent, with a concentration of 15,659.19 μg/g dry extract, followed by quinic acid (9429.91 μg/g dry extract), chlorogenic acid (6049.85 μg/g dry extract), narcissin (5374.22 μg/g dry extract), and vicenin 2 (4042.93 μg/g dry extract). Other relatively abundant compounds included nicotiflorin (1770.08 μg/g dry extract), isoquercitrin (1139.76 μg/g dry extract), beta-resorcylic acid (836.84 μg/g dry extract), salicylic acid (568.24 μg/g dry extract), and protocatechuic acid (550.10 μg/g dry extract).

Several phenolic acids and flavonoid derivatives were detected at moderate levels, including vanillic acid (347.04 μg/g dry extract), ferulic acid (222.85 μg/g dry extract), afzelin (201.05 μg/g dry extract), quercetin (145.83 μg/g dry extract), quercetin 3-rutinoside-7-glucoside (117.80 μg/g dry extract), caffeic acid (106.36 μg/g dry extract), astragalin (103.74 μg/g dry extract), protocatechuic aldehyde (88.08 μg/g dry extract), isorhamnetin (83.54 μg/g dry extract), isoorientin (82.58 μg/g dry extract), and orientin (81.75 μg/g dry extract). Lower amounts of gallic acid, ellagic acid, trans-cinnamic acid, fisetin hydrate, naringenin, vitexin, 3-hydroxyphenylacetic acid, and diosmetin were also detected.

In contrast, several target compounds, including 4-hydroxybenzoic acid, 3-hydroxybenzoic acid, vanillin, coumaric acid, sinapic acid, catechin, apigenin, acacetin, genkwanin, schaftoside, luteolin, luteolin 7-rutinoside, kaempferol, oleuropein, and emodin, were not detected under the applied analytical conditions. Overall, the LC–MS/MS profile indicated that the extract was particularly rich in flavonoid glycosides and phenolic acids.

**Table 3:** Retention times, MRM transitions, and analytical validation parameters (R^2^, LOD, LOQ, and recovery) for the LC–MS/MS quantification of phytochemical standards in the extract.

Compounds	RT	Parent Ion (*m*/*z*)	Fragment Ions (*m*/*z*)	R^2^	LOD (ng/mL)	LOQ (ng/mL)	Recovery (%)
4-Hydroxybenzoic acid	7.82	137.02442	93.03471	0.9921	1.03	3.44	104.89
Salicylic acid	10.93	137.02442	93.03468	0.9946	1.16	3.87	105.31
3-hydroxybenzoic acid	8.66	137.02442	93.03471	0.9926	1.71	5.70	103.14
3-hydroxyphenylaceticacid	8.67	151.04007	107.05045	0.9923	2.62	8.73	104.09
Gallic acid	3.70	169.01425	125.02461	0.9903	2.94	9.78	105.96
Protocatechuic acid	6.24	153.01933	109.02949	0.9908	0.73	2.44	103.64
Protocatechuic aldehyde	7.29	137.02442	136.01671	0.9909	0.46	1.55	101.52
beta-Resorcylic acid	8.42	153.01933	67.01888	0.9935	0.84	2.79	99.39
Vanillic acid	8.57	167.03498	108.02173	0.9938	1.87	6.24	101.12
Vanillin	9.23	151.04007	108.02178	0.9913	1.61	5.37	104.71
trans-Cinnamic acid	12.31	147.04515	47.04515	0.9952	2.25	7.52	102.50
Coumaric acid	9.80	163.04007	119.05027	0.9928	1.01	3.37	100.41
Caffeic acid	8.62	179.03498	135.04509	0.9917	0.63	2.09	103.81
Ferulic acid	10.05	193.05063	134.03751	0.9933	1.39	4.63	101.37
Sinapic acid	10.02	223.06120	193.01436	0.9934	1.03	3.44	101.24
Chlorogenic acid	8.07	353.08781	191.05624	0.9976	0.80	2.65	101.66
Quinic acid	0.93	191.05611	85.02962	0.9939	0.80	2.68	101.67
Catechin	7.62	289.07176	109.02975	0.9943	0.66	2.20	108.18
Apigenin	13.24	269.04555	117.03464	0.9932	1.05	3.51	100.61
Acacetin	14.93	283.06120	268.03717	0.9911	1.92	6.41	101.74
Vicenin 2	9.05	593.15119	473.10941	0.9945	1.09	3.63	108.34
Genkwanin	14.93	283.06120	268.03745	0.9911	1.31	4.37	102.44
Vitexin	10.09	431.09837	311.05603	0.9939	0.92	3.06	109.65
Schaftoside	9.66	563.14063	443.09949	0.9908	2.01	6.69	107.79
Rutin hydrate	10.67	609.14611	300.02777	0.9912	1.28	4.28	103.79
Luteolin	12.53	285.04046	175.04039	0.9932	1.54	5.12	98.29
Orientin+ Isoorientin	9.76	447.09328	327.05148	0.9955	0.88	2.95	107.17
Luteolin 7-rutinoside	10.40	593.15119	285.04065	0.9923	0.70	2.33	108.50
Isoquercitrin	10.74	463.08820	300.02768	0.9939	0.70	2.32	105.86
Narcissin	11.41	623.16176	315.05139	0.9918	1.26	4.21	107.64
Quercetin 3-rutinoside-7-glucoside	11.53	287.05536	135.04512	0.9934	0.76	2.53	105.23
Isorhamnetin	13.21	315.05103	300.02780	0.9958	0.59	1.95	107.06
Kaempferol	13.08	285.04046	136.01686	0.9924	0.57	1.91	99.98
Afzelin	11.98	431.09837	285.04028	0.9951	1.96	6.53	103.95
Nicotiflorin	11.31	593.15119	285.04041	0.9915	0.75	2.49	105.07
Astragalin	11.34	447.09328	284.03281	0.9936	1.51	5.02	105.97
Fisetin hydrate	11.48	285.04046	135.00900	0.9913	1.21	4.03	105.85
Naringenin	12.34	271.06120	119.05035	0.9932	1.46	4.85	99.44
Oleuropein	11.78	542.16678	395.07828	0.9929	1.96	6.53	100.84
Ellagic acid	10.93	300.99899	300.99872	0.9943	0.68	2.27	101.4
Emodin	16.35	269.04555	225.05573	0.9909	1.34	4.46	101.65

RT: retention time, LOD: limit of detection, LOQ: limit of quantification, and R^2^: coefficient of determination.

### 2.3. Fatty Acid Composition

The fatty acid composition of *E. tenuifolia* subsp. *sibthorpiana* oil determined by GC-FID analysis is presented in Table 4. Among the identified fatty acids, lauric acid (C12:0) was the predominant component, accounting for 29.71% of the total fatty acids. Other major constituents were docosadienoic acid (C22:2) at 9.68%, palmitoleic acid (C16:1) at 8.39%, caprylic acid (C8:0) at 8.37%, lignoceric acid (C24:0) at 5.52%, EPA (C20:5) at 4.30%, and caproic acid (C6:0) at 4.11%.

Moderate amounts were observed for undecylic acid (C11:0) (2.76%), pentadecenoic acid (C15:1) (2.43%), tricosanoic acid (C23:0) (2.15%), *cis*-11-eicosenoic acid (C20:1) (2.06%), α-linolenic acid (C18:3 n3) (2.04%), and arachidonic acid (C20:4) (2.02%). The remaining fatty acids were detected at levels below 2%, including capric, tridecylic, myristic, pentadecylic, palmitic, stearic, oleic/elaidic, linoleic/linolelaidic, γ-linolenic, arachidic, and erucic acids.

Overall, the fatty acid profile of the oil was characterized by the predominance of lauric acid together with appreciable amounts of selected medium-chain, long-chain, and polyunsaturated fatty acids.

### 2.4. Volatile Compound Profile

The volatile compound composition of the aerial parts of *E. tenuifolia* subsp. *sibthorpiana* identified by GC–MS is presented in Table 5. A total of 39 compounds were identified. The volatile profile was dominated by methyl eugenol (51.10%), followed by Δ-3-carene (32.23%), indicating that these two compounds constituted the major proportion of the detected volatile fraction. Other relatively abundant constituents were α-phellandrene (3.07%), terpinolene (2.13%), limonene (1.67%), α-terpineol (1.26%), p-cymen-8-ol (0.89%), and p-cymene (0.87%).

In addition to these major constituents, several compounds were detected at lower levels, including (−)-α-pinene (0.67%), γ-terpinene (0.52%), myristicin (0.50%), palmitic acid (0.50%), carvacrol (0.48%), elemicin (0.44%), eucarvone (0.39%), p-cymenene (0.38%), phytol (0.35%), and myristic acid (0.27%). The remaining compounds were present at proportions below 0.25%.

According to their chemical classes, the volatile fraction consisted mainly of monoterpenes and phenylpropanoids, together with smaller amounts of sesquiterpenes, oxygenated monoterpenes, esters, ketones, fatty acids, diterpenes, diterpene alcohols, and other minor constituents. Overall, the GC–MS results showed that the volatile profile of *E. tenuifolia* subsp. *sibthorpiana* was characterized predominantly by methyl eugenol and Δ-3-carene.

### 2.5. Cytotoxic Activity

**Figure 1:**
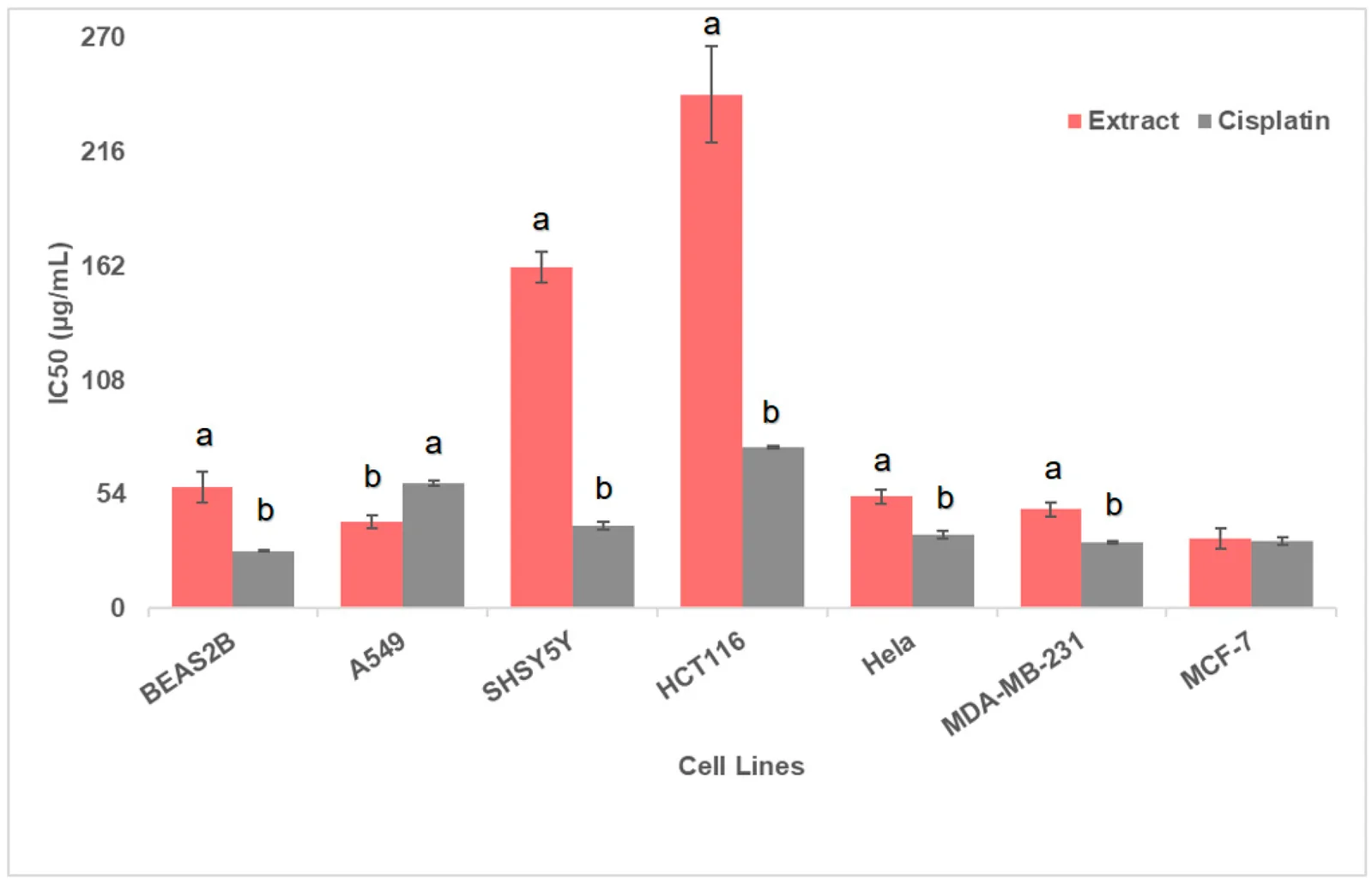
The cytotoxic effects of the extract and cisplatin on healthy and cancerous cells (BEAS-2B, A549, SH-SY5Y, HCT116, HeLa, MDA-MB-231, MCF-7). The bar plots represent the mean IC_50_ values (µg/mL) ± standard deviation (SD) from three independent experiments. Different letters indicate statistically significant differences according to one-way ANOVA followed by Tukey’s post hoc test *(p* < 0.05).

The cytotoxic effects of extract on human cancer cell lines and the non-cancerous BEAS-2B cell line were evaluated using the MTT assay, and the results are presented in Figure 1. The extract exhibited variable cytotoxic effects across the tested cell lines. The lowest IC_50_ value was observed for MCF-7 cells (32.96 ± 4.80 µg/mL), followed by A549 (40.87 ± 3.11 µg/mL), MDA-MB-231 (47.02 ± 3.43 µg/mL), HeLa (53.02 ± 3.50 µg/mL), and BEAS-2B (57.47 ± 7.34 µg/mL) cells. In contrast, the extract showed weaker cytotoxic effects against SH-SY5Y (161.61 ± 7.30 µg/mL) and HCT116 (243.31 ± 22.58 µg/mL) cells.

For comparison, the reference drug cisplatin demonstrated stronger cytotoxic activity across most of the tested cell lines, with IC_50_ values of 27.30 ± 0.51 µg/mL for BEAS-2B, 59.51 ± 1.20 µg/mL for A549, 39.16 ± 1.91 µg/mL for SH-SY5Y, 76.33 ± 0.59 µg/mL for HCT116, 35.06 ± 1.76 µg/mL for HeLa, 31.44 ± 0.59 µg/mL for MDA-MB-231, and 31.79 ± 1.90 µg/mL for MCF-7 cells. Overall, the extract exhibited moderate cytotoxic activity, particularly against MCF-7, A549, and MDA-MB-231 cell lines (Figure 1).

### 2.6. Flow Cytometric Evaluation of Apoptosis

The extract significantly increased apoptosis in all tested cell lines compared with the untreated controls (*p* < 0.05; Table 6). The response was cell-line dependent, with the highest apoptotic rate observed in MCF-7 cells (57.93 ± 1.80%), whereas HCT116 cells (6.00 ± 0.17%) showed the lowest sensitivity. Cisplatin induced significantly greater apoptosis than the extract in all cell lines. Representative Annexin V-FITC/PI flow cytometry plots are provided in Supplementary Figure S4.

### 2.7. Effects on Cell Cycle Distribution

Treatment with the extract significantly altered cell cycle distribution in all tested cell lines compared with the untreated controls (*p* < 0.05; Table 7). The predominant effect was an accumulation of cells in the G2/M phase accompanied by a reduction in the S phase. This shift was particularly evident in MCF-7 cells, where the G2/M population increased from 14.57 ± 0.12% to 34.27 ± 0.67%, and in HCT116 cells, where it increased from 10.53 ± 0.31% to 37.37 ± 0.67%. These findings indicate that the extract induced a pronounced G2/M-phase accumulation, although the magnitude of the response varied among cell lines. Representative DNA-content histograms are presented in Supplementary Figure S5.

### 2.8. Antimicrobial Activity

The antimicrobial activity of the extract was evaluated by disc diffusion and broth microdilution assays, and the results are presented in Table 8. The extract showed a strain-dependent activity profile, with the strongest effect observed against *S. aureus*, which exhibited the largest inhibition zone (14.10 ± 0.15 mm) and the lowest MIC value (400 µg/mL). Lower activity was observed against *C. albicans*, *C. glabrata*, *E. coli*, and *E. faecalis*, with MIC values of 800 µg/mL, whereas *P. aeruginosa* was not inhibited within the tested concentration range. As expected, caspofungin and ampicillin exhibited substantially greater activity than the extract against the corresponding fungal and bacterial strains. Overall, the findings indicate selective but relatively weak antimicrobial activity of the extract.

## 3. Discussion

The present study provides an integrated characterization of *E. tenuifolia* subsp. *sibthorpiana* by combining phytochemical profiling with the evaluation of radical scavenging, cytotoxic, apoptosis-inducing, cell cycle-modulating, and antimicrobial activities. Although previous studies have examined selected biological properties or essential oil compositions of *Echinophora* species, studies integrating detailed LC–MS/MS-based phenolic profiling, GC–MS volatile analysis, GC-FID fatty acid characterization, mechanistic anticancer evaluation, and quantitative antimicrobial assessment within a single framework remain limited. In this respect, the present findings contribute to a broader understanding of the chemical composition and biological profile of *E. tenuifolia* subsp. *sibthorpiana* and support its relevance as a source of natural bioactive compounds.

The high total phenolic content and strong radical scavenging activity observed in the present study indicate that *E. tenuifolia* subsp. *sibthorpiana* is a rich source of antioxidant constituents. The extract exhibited a TPC value of 181.78 ± 5.03 mg GAE/g, together with marked radical scavenging activity in both DPPH (333.65 ± 2.94 mg TE/g) and ABTS (262.18 ± 6.80 mg TE/g) assays. Compared with other *Echinophora* species, the phenolic content of the present extract appears relatively high. For example, the methanolic extract of *E. chrysantha* was reported to contain 325.72 ± 0.86 μg GAE/g (0.325 mg GAE/g) [12], whereas *E. tournefortii* exhibited a TPC value of 159.05 ± 3.42 mg GAE/g [1]. In another study, *E. tenuifolia* subsp. *sibthorpiana* extracts obtained using ethyl acetate and acetone showed TPC values of 141.6 and 46.97 μg GAE/mL, respectively, together with high DPPH inhibition at elevated concentrations [14]. Beyond the genus *Echinophora*, phenolic-rich extracts from other medicinal and edible materials, such as *Sargassum pallidum*, have also been reported to exhibit marked DPPH and ABTS radical scavenging activities [26]. These differences may reflect variations in extraction solvent, plant material, and experimental conditions, which can affect phenolic recovery and radical scavenging activity. The pronounced radical scavenging activity observed here is likely associated with the high abundance of phenolic acids and flavonoid glycosides identified by LC–MS/MS, particularly chlorogenic acid, rutin hydrate, nicotiflorin, and isoquercitrin, together with the high level of quinic acid. These constituents may contribute to radical scavenging through hydrogen- or electron-donating mechanisms [27]. Taken together, the antioxidant findings support the view that the phenolic-rich composition of *E. tenuifolia* subsp. *sibthorpiana* is a major determinant of its bioactive potential.

The LC–MS/MS analysis further demonstrated that *E. tenuifolia* subsp. *sibthorpiana* possesses a phytochemical profile dominated by phenolic acids and flavonoid glycosides, consistent with the general phytochemical pattern reported for medicinal and aromatic plants [4,13,28,29,30,31,32]. Among the detected compounds, rutin hydrate, quinic acid, chlorogenic acid, narcissin, vicenin 2, nicotiflorin, and isoquercitrin were the predominant constituents. Importantly, previous studies on *E. tenuifolia* subsp. *sibthorpiana* have also reported rutin and chlorogenic acid among the major phenolic constituents, together with p-coumaric, ferulic, salicylic, and rosmarinic acids [33,34]. These findings are broadly consistent with the phenolic profile observed in the present study. Because detailed LC–MS/MS-based phenolic characterization of *E. tenuifolia* subsp. *sibthorpiana* remains relatively limited, comparisons with other *Echinophora* species were also considered where relevant. In *E. chrysantha*, RP-HPLC analysis of methanolic extracts from different plant parts identified chlorogenic acid, quercetin, hesperidin, rosmarinic acid, and epicatechin among the major phenolic constituents [4]. Similarly, LC–MS/MS analysis of the hydroalcoholic extract of *E. chrysantha* revealed quinic acid (44.17 mg/g extract), chlorogenic acid (15.85 mg/g extract), rutin (31.48 mg/g extract), quercetin-3-O-glucoside (29.41 mg/g extract), and hesperidin (11.05 mg/g extract) as major compounds [35]. The predominance of rutin- and chlorogenic acid-related compounds in the present study therefore agrees with the broader phytochemical pattern described for the genus. These metabolites are widely recognized for their antioxidant properties [1,14,32,36], which is consistent with the strong radical scavenging activity observed here. In addition, quercetin derivatives, naringenin, and ferulic acid have been associated with antimicrobial and antiproliferative effects [4,37]. Although these constituents were detected at lower levels than rutin hydrate or quinic acid, they may contribute collectively to the observed biological effects through additive or synergistic interactions. Comparative evidence from *E. tenuifolia* subsp. *sibthorpiana* used in tarhana production, where gallic acid, (+)-catechin, and 1,2-dihydroxybenzene were identified as major phenolics [30], as well as from *E. platyloba*, in which quercetin, myricetin, and luteolin were quantified [3,36], further indicates that phenolic composition may vary markedly depending on species, plant material, extraction conditions, and analytical methodology. Accordingly, the present extract appears to be particularly enriched in flavonoid glycosides rather than simple aglycones, a feature that may contribute to its pronounced radical scavenging activity and, together with other constituents, to its cytotoxic, apoptosis-inducing, cell cycle-modulating, and strain-dependent antimicrobial effects.

The volatile profile of *E. tenuifolia* subsp. *sibthorpiana* was dominated by methyl eugenol (51.10%) and Δ-3-carene (32.23%), followed by α-phellandrene (3.07%) and several minor monoterpenes and phenylpropanoid derivatives. Volatile compounds play important ecological roles in plants and have been associated with several biological effects relevant to human health, including antimicrobial, antioxidant, and anti-inflammatory activities [6,11,30,31,32]. The predominance of methyl eugenol observed in the present study is consistent with previous reports on *E. tenuifolia* subsp. *sibthorpiana*, in which this compound has been identified as a major constituent at proportions ranging from 9.61% to 90.16%, depending on geographic origin, developmental stage, and processing conditions [3,11]. α-Phellandrene and p-cymene have also been reported among the characteristic constituents of this species [3,11]. Thus, the present findings fall within the range of previously reported variation and further support methyl eugenol as a characteristic component of this taxon. Comparisons with other *Echinophora* species also indicate considerable interspecific variability in volatile composition. For example, *E. chrysantha* has been reported to contain p-cymene (20.63%), α-phellandrene (9.42%), and Δ-3-carene (8.78%) as major constituents [12], whereas *E. cinerea* and *E. platyloba* are generally characterized by monoterpene-rich profiles containing high levels of α-phellandrene, α-pinene, and β-ocimene [3,36,38,39]. Relative to these species, the high proportion of methyl eugenol detected in *E. tenuifolia* subsp. *sibthorpiana* may indicate a distinct chemotype. Methyl eugenol and other phenylpropanoids have been associated with antimicrobial and antioxidant effects, while monoterpenes such as α-phellandrene and limonene have also been linked to antimicrobial and anti-inflammatory activities [3,6,11,32]. However, the essential oil and the acidified methanolic extract represent chemically distinct fractions. Since all radical scavenging, antimicrobial, and cytotoxic assays in the present study were performed using the methanolic extract, the observed biological activities should primarily be interpreted in relation to the constituents identified in that extract. The biological relevance of methyl eugenol, Δ-3-carene, and other essential-oil constituents therefore cannot be directly inferred from the present assays and requires separate experimental evaluation of the essential oil.

The fatty acid analysis revealed a chemically diverse lipophilic profile dominated by lauric acid (29.71%), followed by docosadienoic acid (9.68%), palmitoleic acid (8.39%), caprylic acid (8.37%), lignoceric acid (5.52%), and eicosapentaenoic acid (EPA, 4.30%). Previous studies have also identified lipid-related constituents in other *Echinophora* species, although the reported profiles vary considerably. In *E. tenuifolia* subsp. *sibthorpiana*, methyl palmitate (1.0%) and methyl elaidate (1.3%) were previously detected in the n-hexane fraction, while myristic acid (7.0%) and palmitic acid (5.9%) were reported among the major constituents [40]. In contrast, lauric acid was the predominant fatty acid in the present study, whereas myristic and palmitic acids occurred at substantially lower levels. Similarly, methyl 2, 5-octadecadienoate has been reported in *E. platyloba* [29], while methyl linoleate was identified in *E. orientalis* [10], further supporting marked interspecific variation in lipid composition. Although lauric acid and other saturated and unsaturated fatty acids have been associated with membrane-related and inflammatory processes [22,40], the biological activity of the separately obtained oil fraction was not evaluated in the present study. Therefore, the fatty acid data primarily provide complementary chemotaxonomic and compositional information rather than direct mechanistic evidence for the activities observed in the methanolic extract.

The cytotoxic evaluation demonstrated that *E. tenuifolia* subsp. *sibthorpiana* exerted moderate but measurable effects across a panel of human cancer cell lines. The extract was most active against MCF-7 (32.96 ± 4.80 µg/mL), A549 (40.87 ± 3.11 µg/mL), and MDA-MB-231 (47.02 ± 3.43 µg/mL) cells, whereas weaker effects were observed against SH-SY5Y (161.61 ± 7.30 µg/mL) and HCT116 (243.31 ± 22.58 µg/mL) cells. The IC_50_ value obtained in the non-cancerous BEAS-2B cell line (57.47 ± 7.34 µg/mL), which was in the same order of magnitude as those observed for MCF-7, A549, and MDA-MB-231 cells, indicates that the extract exhibited limited selectivity toward cancer cells under the present conditions. Previous studies on *Echinophora* species have reported stronger cytotoxic effects in some cases. Methanolic extracts of *E. chrysantha*, for instance, showed IC_50_ values as low as 1.70 and 7.40 µg/mL against HT-29 and A549 cells, respectively [4]. Likewise, dichloromethane fractions of *E. tenuifolia* subsp. *sibthorpiana* were reported to exhibit IC_50_ values of 22.8 µg/mL against C32 melanoma cells and 53.0 µg/mL against LoVo colon adenocarcinoma cells [3,22]. In contrast, *E. platyloba* extracts showed more moderate cytotoxicity but greater selectivity toward cancer cells than normal cells [3,22,23]. Compared with these reports, the cytotoxic effect of the present extract can be considered moderate.

The flow cytometric findings further showed that the reduction in cell viability was accompanied by apoptosis induction and alterations in cell cycle distribution. The extract significantly increased apoptosis in all tested cell lines, with the strongest response observed in MCF-7 cells, which were also among the most sensitive in the MTT assay. In parallel, *E. tenuifolia* subsp. *sibthorpiana* treatment caused a consistent accumulation of cells in the G2/M phase and a reduction in the S phase, indicating G2/M-phase accumulation under the tested conditions. The cell cycle alterations observed at 5 µg/mL should be interpreted as early cell-cycle perturbations occurring at a low, sub-cytotoxic concentration rather than as direct consequences of the cytotoxic effects observed at higher concentrations in the MTT assay. The activity may be related, at least in part, to the phenolic-rich composition of the extract, including rutin, chlorogenic acid, isoquercitrin, and related flavonoids, which have previously been associated with pro-apoptotic and cell cycle-modulating effects in different cancer models [4,41,42]. However, because the extract contains a complex mixture of compounds, the contribution of individual constituents and possible interactions among them cannot be established from the present data. Further fractionation and target-based mechanistic studies are therefore required to identify the compounds responsible and clarify the signaling pathways involved.

The antimicrobial evaluation revealed a strain-dependent and relatively weak activity profile. In the disc diffusion assay, the extract showed the greatest inhibitory effect against *S. aureus* (14.10 ± 0.15 mm), while smaller inhibition zones were observed against *E. coli*, *C. albicans*, and *C. glabrata*. No inhibition zone was detected against *P. aeruginosa* or *E. faecalis*. The broth microdilution results were consistent with this pattern, with the lowest MIC value obtained against *S. aureus* (400 µg/mL), whereas MIC values of 800 µg/mL were determined for *E. coli*, *E. faecalis*, *C. albicans*, and *C. glabrata*. No activity was detected against *P. aeruginosa* within the tested concentration range. Previous studies on *Echinophora* species have consistently shown that antimicrobial activity is often stronger in essential oils than in solvent extracts [3,4,29,36]. In *E. tenuifolia* subsp. *sibthorpiana* essential oil, MIC values as low as 62.5 and 125 µg/mL have been reported against *Bacillus cereus* and *S. aureus*, respectively [3,13,14,43]. Similarly, essential oils and volatile fractions of *E. platyloba* and *E. cinerea* have shown notable antibacterial and antifungal activities [29,32,44]. Compared with these reports, the activity of the present crude extract was substantially weaker, which may reflect differences in extract composition and the greater concentration of hydrophobic antimicrobial constituents in volatile-rich fractions. The higher susceptibility of *S. aureus* is also consistent with the general tendency of Gram-positive bacteria to be more sensitive to plant-derived compounds than Gram-negative bacteria, whose outer membrane may restrict compound penetration [29,36]. The absence of detectable activity against *P. aeruginosa* within the tested range is therefore not unexpected. The observed antimicrobial effects may be associated with the combined contribution of phenolic constituents, which have been linked to membrane disruption, altered permeability, protein denaturation, and interference with microbial enzymatic systems [6,32,36]. However, because individual compounds were not tested separately, the contribution of specific metabolites and possible interactions among them remains to be clarified.

Overall, the findings of this study demonstrate that *E. tenuifolia* subsp. *sibthorpiana* possesses a chemically diverse profile and exhibits multiple biological activities of varying magnitude. Its strong radical scavenging activity appears to be closely associated with the phenolic-rich composition of the methanolic extract. The extract also showed moderate cytotoxic effects accompanied by apoptosis induction and G2/M-phase accumulation, although its limited activity in the non-cancerous BEAS-2B cell line indicates that tumor selectivity was not pronounced. Antimicrobial activity was strain dependent and relatively weak, with the greatest susceptibility observed in *S. aureus*. Taken together, these findings highlight the value of integrating phytochemical characterization with functional and mechanistic bioactivity assays to obtain a more comprehensive understanding of the biological potential of plant extracts.

Limitations of the study: The present study was based on a single extract obtained from one wild population, which may limit the generalizability of the findings. All biological activities were evaluated in vitro, without in vivo validation. The active constituents responsible for the observed effects were not isolated, and their individual contributions or potential synergistic interactions were not examined. In addition, the molecular mechanisms underlying apoptosis and cell cycle alterations were not investigated. The antimicrobial evaluation was limited to inhibition zone and MIC determinations, and the limited selectivity toward cancer cells over BEAS-2B cells should be considered when interpreting the anticancer potential.

## 4. Materials and Methods

### 4.1. Plant Material

In this study, *E. tenuifolia* subsp. *sibthorpiana* was collected from Banazı town, Malatya Province, Türkiye (38.296857° N, 38.291000° E), in August 2023 during the full flowering stage. Plant authentication was performed by Dr. Turgay Kolaç (Pharmacognosist, Department of Pharmacy Services, Inonu University) according to the Flora of Turkey and the East Aegean Islands [45], and a voucher specimen was deposited in the Herbarium of the Faculty of Pharmacy, Inonu University, under the voucher number TK1473. After collection, the plant material was dried under shade in a well-ventilated environment without direct exposure to sunlight. Once completely dried, the aerial parts of the plant, comprising stems, leaves, and flowers, were used together for further analyses, while the roots were excluded. The dried material was ground into a fine powder using a laboratory mill and stored in airtight containers at room temperature, protected from moisture and light, until extraction and subsequent analyses.

### 4.2. Extraction Procedure

The powdered aerial parts of *E. tenuifolia* subsp. *sibthorpiana* were extracted using methanol (Merck, Darmstadt, Germany) acidified with 0.05% HCl. This solvent system was selected to improve the solubility and recovery of phenolic acids and flavonoid glycosides. Mild acidification may facilitate the release of phenolic constituents associated with plant tissues and may contribute to improved extraction efficiency [46]. The mild acidification level was selected to enhance phenolic recovery without compromising compatibility with subsequent LC–MS/MS analysis. For extraction, 10 g of powdered plant material was macerated with 100 mL of acidified methanol (0.05% HCl) at room temperature in the dark for 24 h. The extraction procedure was repeated three times, and the resulting filtrates were combined. The combined extracts were filtered through Whatman No. 1 filter paper, and the solvent was removed under reduced pressure using a rotary evaporator at 40 °C to obtain the crude dry extract. The extraction yield was calculated based on the dry weight of the plant material. A total of 1.125 g of dry extract was obtained from 10 g of powdered aerial parts, corresponding to an extraction yield of 11.25%. The dried extract was stored in amber glass containers at 4 °C, protected from light, until further analysis.

### 4.3. Determination of Total Phenolic Content (TPC)

The TPC was determined using the Folin–Ciocalteu reagent (Merck, Germany) method with minor modifications [47]. Briefly, 0.5 mL of the extract solution was mixed with 2.5 mL of 10% Folin–Ciocalteu reagent. After incubation at room temperature for 5 min, 2 mL of 7.5% sodium carbonate (Na_2_CO_3_) solution was added. The reaction mixture was then incubated in the dark at room temperature for 30 min. Absorbance was measured at 765 nm using a UV-Vis spectrophotometer (Shimadzu 2000S, Kyoto, Japan). A calibration curve was prepared using gallic acid, and the results were expressed as milligrams of gallic acid equivalents per gram of dry extract (mg GAE/g).

### 4.4. Determination of Antioxidant Capacity

#### 4.4.1. DPPH Radical Scavenging Activity

The DPPH radical scavenging activity of the extract was evaluated based on its ability to scavenge the stable DPPH radical (2,2-diphenyl-1-picrylhydrazyl). In brief, 100 µL of the extract was added to 3.9 mL of freshly prepared DPPH (Sigma-Aldrich, St. Louis, MO, USA) solution. The mixture was vortexed and incubated in the dark at room temperature for 30 min. Absorbance was then measured at 517 nm using a UV-Vis spectrophotometer (Shimadzu 2000S, Japan). DPPH radical scavenging activity was quantified using a Trolox (Sigma-Aldrich, USA) calibration curve and expressed as milligrams of Trolox equivalents per gram of dry extract (mg TE/g) [28].

#### 4.4.2. ABTS Radical Scavenging Assay

The ABTS radical scavenging activity of the extract was also evaluated using the ABTS [2,2′-azino-bis (3-ethylbenzothiazoline-6-sulfonic acid)] radical scavenging assay, as described by Zengin et al. [48]. Briefly, 50 µL of the extract was mixed with 150 µL of extraction solvent and 3800 µL of freshly prepared ABTS+• (Sigma-Aldrich, USA) radical solution. The mixture was vortexed and incubated in the dark at room temperature for 15 min. After incubation, absorbance was measured at 734 nm using a UV-Vis spectrophotometer (Shimadzu 2000S, Kyoto, Japan). The ABTS radical scavenging activity was expressed as milligrams of Trolox equivalents per gram of dry extract (mg TE/g).

### 4.5. LC–MS/MS Analysis of Phytochemical Compounds

The phytochemical composition of the extract was analyzed using a Thermo Scientific UHPLC system (Thermo Fisher Scientific Inc., Waltham, MA, USA) coupled to a TSQ Quantum Access MAX triple quadrupole mass spectrometer equipped with an electrospray ionization (ESI) source. Chromatographic separation was performed on an Accucore C18 column (100 mm × 2.1 mm, 2.6 µm; Thermo Scientific, USA) maintained at 30 °C. The mobile phases consisted of ultrapure water containing 0.1% (*v/v*) formic acid (solvent A) and methanol containing 0.1% (*v/v*) formic acid (solvent B). A linear gradient elution program was applied as follows: 0–2 min, 5% B; 2–10 min, 5–95% B; 10–12 min, 95% B; 12–13 min, return to 5% B; followed by re-equilibration until 15 min. The flow rate was 0.3 mL/min, and the injection volume was 10 µL. MS/MS analyses were performed in negative ion mode with the following parameters: spray voltage 3500 V, capillary temperature 320 °C, sheath gas 35 arb, auxiliary gas 10 arb, and collision gas pressure 1.5 mTorr. Target compounds were identified and quantified in multiple-reaction monitoring (MRM) mode by comparing their retention times and precursor/product-ion transitions with those of authentic reference standards. Fragmentation patterns and relevant literature data were used as additional confirmation criteria [49]. For sample preparation, the dried extract was dissolved in LC–MS-grade methanol at a final concentration of 1 mg/mL and filtered through a 0.22 µm PTFE syringe filter (MARZ Shuller, ADGLAB, İstanbul, Türkiye) before injection.

Method validation was carried out using calibration standards prepared for each analyte according to the International Council for Harmonisation (ICH) Q2(R1) guideline [50]. Calibration curves were established in the linear range of 10–100 ng/mL. The coefficients of determination (R^2^) ranged from 0.9903 to 0.9976. The limits of detection (LOD) and limits of quantification (LOQ), calculated at signal-to-noise ratios of 3 and 10, ranged from 0.46 to 2.94 ng/mL and from 1.55 to 9.78 ng/mL, respectively. Recovery values ranged between 98.29% and 109.65%. The MRM transitions, retention times, and method-validation results, including R^2^, LOD, LOQ, and recovery values, are presented in Table 3.

All solvents, reagents, and analytical standards used for the LC–MS/MS analysis were purchased from Sigma-Aldrich (Darmstadt, Germany) and were of analytical or LC–MS grade, as appropriate. A representative LC–MS/MS chromatogram is provided in Supplementary Figure S1.

### 4.6. Oil Extraction and Fatty Acid Composition

The oil was obtained from the aerial parts by Soxhlet extraction using hexane (Merck, Germany) as the extraction solvent. The extracted oil was then used for fatty acid composition analysis after conversion to fatty acid methyl esters (FAMEs). For this purpose, 20–40 mg of oil was mixed with 9.5 mL hexane and 0.5 mL of 2 M methanolic KOH, shaken vigorously for 30 s, and kept in the dark at room temperature for 1 h. An aliquot of the clear upper phase was then injected into the GC-FID system [51]. Analysis was performed on an Agilent gas chromatograph (Agilent Technologies, Santa Clara, CA, USA) equipped with an FID detector and a TR-CN100 capillary column (60 m × 0.25 mm, 0.20 µm) (Teknokroma, Sant Cugat del Vallès, Barcelona, Spain). The injector temperature was set at 240 °C, the split ratio was 1:100, and helium was used as the carrier gas at a flow rate of 1 mL/min. The oven temperature was initially held at 90 °C for 7 min and then increased to 240 °C at a rate of 5 °C/min. The injection volume was 1 µL. Fatty acids were identified by comparing their retention times with those of standard mixtures, and the results were expressed as percentage composition. A representative GC–FID chromatogram is provided in Supplementary Figure S2.

### 4.7. Hydrodistillation and GC–MS Analysis of Essential Oil

Essential oil was obtained from the aerial parts of the plant by hydrodistillation using a Clevenger-type apparatus. Briefly, 100 g of plant material was placed in a 1000 mL round-bottom flask, and 300 mL of distilled water was added. The mixture was subjected to hydrodistillation for 2.5 h. Hydrodistillation yielded 0.52 mL of essential oil from 100 g of dried plant material, corresponding to a yield of 0.52% (*v/w*). The collected essential oil was separated from the aqueous phase and stored in a cool, dark environment until GC–MS analysis. GC–MS analysis was performed using an Agilent 6890N Network GC System coupled with an Agilent 5973 Inert Mass Selective Detector (MSD) (Agilent Technologies, USA), equipped with a TRB-WAX capillary column (30 m × 0.25 mm, 0.25 µm film thickness; Teknokroma, Spain). Helium was used as the carrier gas at a flow rate of 1 mL/min. A 1 µL aliquot was injected in splitless mode, and the injector temperature was maintained at 250 °C. The oven temperature was programmed from 50 °C to 160 °C at a rate of 3 °C/min and held for 10 min, and then increased to 260 °C at the same rate. Mass spectra were recorded under electron ionization at 70 eV over an m/z range of 35–450. Relative retention indices were calculated using a homologous series of n-alkanes (C8–C35) analyzed under comparable chromatographic conditions. The obtained values were used as supportive criteria together with mass spectral library matching for compound identification. Compounds were identified by comparing their mass spectra with those in the NIST mass spectral library. Data acquisition and processing were performed using Agilent ChemStation software D01.01 [52]. A representative GC–MS chromatogram of the essential oil is provided in Supplementary Figure S3.

### 4.8. Cytotoxic Activity Assay

The cytotoxic activity of extract was evaluated by the MTT assay against a panel of human cell lines, including MDA-MB-231 and MCF-7 (breast adenocarcinoma), A549 (lung carcinoma), SH-SY5Y (neuroblastoma), HCT116 (colorectal carcinoma), HeLa (cervical carcinoma), and the non-cancerous BEAS-2B (human bronchial epithelial) cell line [53]. All cell lines were obtained from the American Type Culture Collection (ATCC, Manassas, VA, USA). Cells were cultured in DMEM (Thermo Fisher, USA) supplemented with 10% fetal bovine serum (Thermo Fisher, USA) and 1% penicillin-streptomycin (Thermo Fisher, USA) at 37 °C in a humidified atmosphere containing 5% CO_2_. For the assay, cells were seeded into 96-well plates at a density of 1 × 10^4^ cells/well and incubated for 24 h. The medium was then replaced with fresh medium containing the extract at concentrations ranging from 0.78 to 200 µg/mL, followed by incubation for an additional 24 h. After treatment, the medium was removed and replaced with MTT (Sigma-Aldrich, USA) solution (0.5 mg/mL), and the cells were incubated for 4 h at 37 °C. The resulting formazan crystals were dissolved in DMSO (Merck, Germany), and absorbance was measured at 570 nm with a reference wavelength of 650 nm using a microplate reader (Epoch, Biotek Instruments, Winooski, VT, USA). Cell viability was expressed as a percentage relative to untreated control cells, and IC_50_ values were calculated from the dose–response curves.

### 4.9. Flow Cytometric Apoptosis Analysis

Apoptotic changes in the plasma membrane were evaluated after treatment with the extract at 50 µg/mL for 24 h using an Annexin V-FITC/PI Apoptosis Detection Kit (E-CK-A211, Elabscience, Houston, TX, USA), according to the manufacturer’s instructions. Briefly, cells were harvested, transferred into 12 × 75 mm polystyrene tubes, and centrifuged at 1100 rpm for 5 min at room temperature. The resulting cell pellets were resuspended in 1–2 mL of Annexin V Binding Buffer (Elabscience, Wuhan, China) and centrifuged again under the same conditions. After removal of the supernatant, each pellet was resuspended in 100 µL of propidium iodide (PI) (Elabscience, Wuhan, China) working solution prepared in Annexin V Binding Buffer, followed by the addition of 5 µL of Annexin V-FITC. The samples were incubated for 15 min at room temperature in the dark. Subsequently, 400 µL of Annexin V Binding Buffer was added to each tube, and the samples were maintained on ice until flow cytometric analysis. Stained cells were analyzed using a BD Accuri C6 Plus flow cytometer (BD Biosciences, San Jose, CA, USA). Based on Annexin V-FITC and PI staining patterns, cell populations were classified as viable (Annexin V^−^/PI^−^), early apoptotic (Annexin V^+^/PI^−^), late apoptotic or secondary necrotic (Annexin V^+^/PI^+^), and necrotic (Annexin V^−^/PI^+^) [54].

### 4.10. Cell Cycle Analysis by Propidium Iodide Staining

The effects of the extract and cisplatin on cell cycle distribution were evaluated as previously described [55]. Briefly, cells were treated with the extract or cisplatin at a concentration of 5 µg/mL for 24 h. This concentration was selected as a sub-cytotoxic dose to evaluate early alterations in cell cycle progression while minimizing the confounding effects of extensive cell death. The cells were subsequently fixed in 70% ethanol. Approximately 10^6^–10^7^ cells were collected, resuspended in 5 mL of phosphate-buffered saline (PBS), and centrifuged at approximately 200× *g* for 6 min. The resulting pellet was gently resuspended in 0.5 mL of PBS to obtain a single-cell suspension and minimize cell aggregation during fixation. The cell suspension was then transferred dropwise into tubes containing cold 70% ethanol and incubated at 4 °C for at least 2 h. Following fixation, the cells were centrifuged, washed with PBS, and resuspended in 1 mL of propidium iodide solution containing RNase A. The samples were incubated for 15 min at 37 °C or 30 min at room temperature in the dark. Cell cycle distribution was analyzed using a BD Accuri C6 Plus flow cytometer, and the proportions of cells in the G0/G1, S, and G2/M phases were determined.

### 4.11. Antimicrobial Activity Assays

#### 4.11.1. Disc Diffusion Assay

The antibacterial and antifungal activities of the extract were evaluated using the disc diffusion method [46]. The tested microorganisms were *Enterococcus faecalis* (ATCC 29212), *E. coli* (ATCC 25922), *S. aureus* (ATCC 29213), *Pseudomonas aeruginosa* (ATCC 27853), *C. albicans* (SC5314/ATCC MYA-2876), and *Candida glabrata* (ATCC 2001). The extract was dissolved in 100% DMSO at 10 µg/µL, and 100 µL was loaded onto sterile 6-mm paper discs. Bacterial and yeast suspensions were prepared at approximately 1 × 10^8^ and 1 × 10^7^ CFU/mL, respectively, spread onto agar plates, and incubated with the extract-loaded discs at 37 °C for 24 h. Ampicillin and caspofungin (100 µg/disc) were used as positive controls, while DMSO served as the negative control. Antimicrobial activity was expressed as the inhibition zone diameter in millimeters.

#### 4.11.2. Minimum Inhibitory Concentration Determination

The minimum inhibitory concentration (MIC) of the extract was determined by the broth microdilution method based on EUCAST E.Def 7.4 for yeasts and CLSI M07 for bacteria. The extract was serially diluted in 96-well plates containing YPD (Yeast Peptone Dextrose) broth (Sigma-Aldrich, USA) for yeasts or LB (Luria-Bertani) broth (Sigma-Aldrich, USA) for bacteria to obtain final concentrations of 0.8–800 µg/mL. The final inoculum densities were 0.5–2.5 × 10^5^ CFU/mL for yeasts and 5 × 10^5^ CFU/mL for bacteria. Plates were incubated at 37 °C for 24 h for yeasts and 16–18 h for bacteria. Yeast growth was measured spectrophotometrically at 530 nm, whereas bacterial growth was evaluated visually. The MIC was defined as the lowest concentration causing at least 50% growth inhibition in yeasts and complete inhibition of visible growth in bacteria [56,57].

### 4.12. Statistical Analysis

All experiments were performed in three independent replicates (n = 3), and results are expressed as the mean ± standard deviation (SD). Statistical analyses were performed using JMP version 7.0 software (SAS Institute Inc., Cary, NC, USA). Comparisons between two groups were performed using Student’s *t*-test, whereas comparisons involving multiple groups were analyzed using one-way analysis of variance (ANOVA) followed by Tukey’s post hoc test. Differences were considered statistically significant at *p* < 0.05.

## 5. Conclusions

This study provides an integrated evaluation of the phytochemical composition and biological activities of *E. tenuifolia* subsp. *sibthorpiana* by combining LC–MS/MS, GC–MS, and GC-FID analyses with complementary in vitro assays. The methanolic extract contained high levels of phenolic acids and flavonoid glycosides, particularly rutin hydrate, quinic acid, chlorogenic acid, narcissin, and vicenin 2, and exhibited pronounced radical-scavenging activity. The essential oil, dominated by methyl eugenol and Δ-3-carene, and the fatty acid profile, characterized by a high proportion of lauric acid, further demonstrate the chemical diversity of this taxon. The extract also showed moderate cytotoxic effects, particularly against MCF-7, A549, and MDA-MB-231 cells. Flow cytometric analyses showed that these effects were accompanied by increased apoptosis and a consistent accumulation of cells in the G2/M phase, indicating the involvement of apoptosis and G2/M-phase accumulation in the cytotoxic response. However, the response observed in the non-cancerous BEAS-2B cell line indicates that cancer-cell selectivity was limited under the tested conditions. The antimicrobial activity of the extract was strain dependent and relatively weak, with S. aureus showing the greatest susceptibility in both disc diffusion and broth microdilution assays. Considering the relatively high MIC values observed for most tested microorganisms, the antimicrobial activity of the extract appears to be limited under the present experimental conditions and should primarily be regarded as preliminary evidence warranting further investigation rather than direct therapeutic applicability.

Overall, the findings identify *E. tenuifolia* subsp. *sibthorpiana* as a chemically diverse source of natural bioactive compounds with strong radical scavenging activity and measurable cytotoxic, apoptosis-inducing, cell cycle-modulating, and antimicrobial effects. Further studies involving bioactivity-guided fractionation, identification of active constituents, molecular pathway analyses, evaluation of cancer-cell selectivity, and in vivo validation are required to clarify its pharmacological relevance and safety.

## Figures and Tables

**Table 1:** TPC and radical scavenging activity of the extract.

	mg GAE/g	mg TE/g	
	TPC	DPPH	ABTS
Extract	181.78 ± 5.03	333.65 ± 2.94	262.18 ± 6.80

Values are expressed as the mean ± standard deviation (SD) of three independent measurements. TPC is expressed as mg gallic acid equivalents per g dry extract (mg GAE/g), whereas DPPH and ABTS radical scavenging activities are expressed as mg Trolox equivalents per g dry extract (mg TE/g).

**Table 2:** Phytochemical compounds identified and quantified in the extract by LC–MS/MS analysis.

Compounds	μg/g Dry Extract	Compounds	μg/g Dry Extract
4-Hydroxybenzoic acid	nd	Vitexin	9.55
Salicylic acid	568.24	Schaftoside	nd
3-hydroxybenzoic acid	nd	Rutin hydrate	15,659.19
3-hydroxyphenylacetic acid	7.50	Luteolin	nd
Gallic acid	44.30	Diosmetin	3.50
Protocatechuic acid	550.10	Orientin	81.75
Protocatechuic aldehyde	88.08	Isoorientin	82.58
beta-Resorcylic acid	836.84	Luteolin 7-rutinoside	nd
Vanillic acid	347.04	Quercetin	145.83
Vanillin	nd	Isoquercitrin	1139.76
trans-Cinnamic acid	36.85	Narcissin	5374.22
Coumaric acid	nd	Quercetin 3-rutinoside-7-glucoside	117.80
Caffeic acid	106.36	Isorhamnetin	83.54
Ferulic acid	222.85	Kaempferol	nd
Sinapic acid	nd	Afzelin	201.05
Chlorogenic acid	6049.85	Nicotiflorin	1770.08
Quinic acid	9429.91	Astragalin	103.74
Catechin	nd	Fisetin hydrate	13.82
Apigenin	nd	Naringenin	12.20
Acacetin	nd	Oleuropein	nd
Vicenin 2	4042.93	Ellagic acid	42.09
Genkwanin	nd	Emodin	nd

Phytochemical compounds were identified and quantified by LC–MS/MS, and the results are expressed as μg/g dry extract. nd: not detected.

**Table 4:** Fatty acid profile of the oil obtained from *E. tenuifolia* subsp. *sibthorpiana* as determined by GC-FID.

Name	Abbreviation	RT (min)	Peak Area	% Area
Caproic acid	C6:0	4.32	1,865,762	4.11
Caprylic acid	C8:0	5.77	3,802,837	8.37
Capric acid	C10:0	6.99	476,804	1.05
Undecylic acid	C11:0	7.64	1,255,089	2.76
Lauric acid	C12:0	8.26	13,497,566	29.71
Tridecylic acid	C13:0	8.97	685,142	1.51
Myristic acid	C14:0	9.70	699,995	1.54
Myristoleic acid	C14:1	10.06	427,283	0.94
Pentadecylic acid	C15:0	10.65	587,885	1.29
Pentadecenoic acid	C15:1	10.99	1,104,295	2.43
Palmitic acid	C16:0	11.75	293,916	0.65
Palmitoleic acid	C16:1	12.10	3,813,665	8.39
Margaric acid	C17:0	12.75	143,605	0.32
Heptadecenoic acid	C17:1	13.37	166,347	0.37
Stearic acid	C18:0	14.40	187,220	0.41
Oleic acid + Elaidic acid	C18:1 c+t	14.74	389,048	0.86
Linoleic acid + Linolelaidic acid	C18:2 c + t	15.44	578,501	1.27
γ-Linolenic acid	C18:3 n6	16.01	198,213	0.44
α-Linolenic acid	C18:3 n3	16.60	925,593	2.04
Arachidic acid	C20:0	17.67	414,173	0.91
cis-11-Eicosenoic acid	C20:1	18.13	934,123	2.06
all cis-11,14-Eicosadienoic acid	C20:2	19.32	390,958	0.86
all cis-8,11,14-Eicosatrienoic acid + Heneicosanoic acid	C20 3n6 + C21:0	19.98	164,455	0.36
all cis-11,14,17-Eicosatrienoic acid	C20:3 n3	20.62	324,281	0.71
Arachidonic acid	C20:4	21.03	918,206	2.02
EPA	C20:5	22.50	1,952,241	4.30
Erucic acid	C22:1	23.52	650,415	1.43
Docosadienoic acid	C22:2	25.55	4,397,595	9.68
Tricosanoic acid	C23:0	26.88	974,666	2.15
Lignoceric acid	C24:0	30.60	2,505,509	5.52
DHA + Nervonic acid	C22:6 + C24:1	31.30	703,317	1.55

Fatty acids were identified by comparison of retention times with those of standard fatty acid methyl ester mixtures and are expressed as relative percentage composition (% area). RT, retention time; Peak Area, integrated chromatographic peak area; % Area, relative percentage of total detected fatty acids; EPA, eicosapentaenoic acid; DHA, docosahexaenoic acid.

**Table 5:** Chemical composition of volatile compounds identified in the aerial parts of *E. tenuifolia* subsp. *sibthorpiana* by GC–MS analysis.

Peak No	RI	Compound	Relative Content (%)	Class	Identification
1	1030	(−)-α-Pinene	0.67	Monoterpene hydrocarbon	RI, MS
2	1132	Sabinene	0.19	Monoterpene hydrocarbon	RI, MS
3	1159	Δ-3-Carene	32.23	Monoterpene hydrocarbon	RI, MS
4	1176	α-Phellandrene	3.07	Monoterpene hydrocarbon	RI, MS
5	1200	Sylvestrene	0.20	Monoterpene hydrocarbon	RI, MS
6	1203	Limonene	1.67	Monoterpene hydrocarbon	RI, MS
7	1214	p-Mentha-1,3,8-triene	0.03	Monoterpene hydrocarbon	RI, MS
8	1231	2-Pentylfuran	0.03	Furan	RI, MS
9	1240	cis-β-Ocimene	0.03	Monoterpene hydrocarbon	RI, MS
10	1255	γ-Terpinene	0.52	Monoterpene hydrocarbon	RI, MS
11	1280	p-Cymene	0.87	Monoterpene hydrocarbon	RI, MS
12	1290	Terpinolene	2.13	Monoterpene hydrocarbon	RI, MS
13	1438	Hexyl 2-methylbutanoate	0.02	Aliphatic Ester	RI, MS
14	1452	p-Cymenene	0.38	Monoterpene hydrocarbon	RI, MS
15	1497	α-Copaene	0.07	Sesquiterpene hydrocarbon	RI, MS
16	1553	Linalool	0.03	Oxygenated monoterpene	RI, MS
17	1571	Eucarvone	0.39	Oxygenated monoterpene	RI, MS
18	1577	cis-4-Caranone	0.08	Oxygenated monoterpene	RI, MS
19	1612	β-Caryophyllene	0.11	Sesquiterpene hydrocarbon	RI, MS
20	1674	α-Phellandren-8-ol	0.08	Oxygenated monoterpene	RI, MS
21	1675	p-Mentha-1,8-dien-4-ol	0.09	Oxygenated monoterpene	RI, MS
22	1702	α-Terpineol	1.26	Oxygenated monoterpene	RI, MS
23	1802	1,3-Cyclohexadiene, 2-methyl- 5-(1-methylethyl)-, monoepoxide	0.18	Oxygenated monoterpene	RI, MS
24	1864	p-Cymen-8-ol	0.89	Oxygenated monoterpene	RI, MS
25	1910	Neophytadiene	0.20	Diterpene hydrocarbon	RI, MS
26	1940	cis-Jasmone	0.21	Other oxygenated compound	RI, MS
27	2023	Methyl eugenol	51.10	Phenylpropanoid	RI, MS
28	2056	Hexyl benzoate	0.07	Benzenoid Ester	RI, MS
29	2113	Cumic alcohol	0.03	Oxygenated monoterpene	RI, MS
30	2120	cis-3-Hexenyl benzoate	0.20	Benzenoid Ester	RI, MS
31	2180	Thymol	0.25	Oxygenated monoterpene	RI, MS
32	2225	Carvacrol	0.48	Oxygenated monoterpene	RI, MS
33	2229	Elemicin	0.44	Phenylpropanoid	RI, MS
34	2258	Myristicin	0.50	Phenylpropanoid	RI, MS
35	2467	Lauric acid	0.09	Fatty acid	RI, MS
36	2603	Phytol	0.35	Oxygenated diterpene	RI, MS
37	2672	Myristic acid	0.27	Fatty acid	RI, MS
38	2822	Pentadecanoic acid	0.11	Fatty acid	RI, MS
39	2890	Palmitic acid	0.50	Fatty acid	RI, MS

Compounds were identified by comparison of their retention indices and mass spectral data with library data. Relative contents are expressed as percentage of total peak area. RI, retention index; MS, identification based on mass spectral data comparison with library spectra.

**Table 6:** Apoptotic cell percentages following treatment with the extract and cisplatin in cancerous and non-cancerous cell lines.

Cell Line	Apoptosis		
	Control	Extract	Cisplatin
BEAS-2B	1.80 ± 0.30 ^c^	20.43 ± 1.94 ^b^	99.50 ± 0.17 ^a^
A549	2.53 ± 0.25 ^c^	27.30 ± 1.40 ^b^	58.10 ± 1.47 ^a^
SH-SY5Y	3.13 ± 0.32 ^c^	10.53 ± 1.52 ^b^	94.93 ± 0.06 ^a^
HCT116	2.23 ± 0.12 ^c^	6.00 ± 0.17 ^b^	58.23 ± 0.21 ^a^
HeLa	1.47 ± 0.29 ^c^	27.23 ± 1.80 ^b^	91.37 ± 1.44 ^a^
MDA-MB-231	3.10 ± 0.26 ^c^	31.53 ± 2.11 ^b^	95.03 ± 1.16 ^a^
MCF-7	3.37 ± 0.06 ^c^	57.93 ± 1.80 ^b^	92.97 ± 0.42 ^a^

Data are presented as the mean ± SD from three independent experiments. Cells were treated with the extract or cisplatin for 24 h, and apoptosis was determined by Annexin V-FITC/PI staining followed by flow cytometric analysis. Different superscript letters within the same row indicate statistically significant differences among treatments according to one-way ANOVA followed by Tukey’s post hoc test (*p* < 0.05).

**Table 7:** Cell cycle distribution (%) in cancerous and non-cancerous cell lines following 24 h treatment with the extract and cisplatin.

Cell Line	Treatment	G0/G1	S	G2/M
BEAS-2B	Control	63.20 ± 0.35 ^a^	21.33 ± 0.40 ^b^	13.03 ± 0.12 ^b^
	Extract	58.77 ± 0.68 ^c^	14.77 ± 0.25 ^c^	22.77 ± 0.47 ^a^
	Cisplatin	55.23 ± 0.25 ^b^	28.80 ± 0.35 ^a^	13.43 ± 0.40 ^b^
A549	Control	85.73 ± 1.11 ^a^	6.87 ± 0.25 ^c^	3.27 ± 0.42 ^b^
	Extract	72.60 ± 0.53 ^c^	10.00 ± 0.20 ^b^	16.43 ± 0.51 ^a^
	Cisplatin	77.50 ± 0.46 ^b^	16.90 ± 0.40 ^a^	2.80 ± 0.26 ^b^
SH-SY5Y	Control	58.43 ± 0.51 ^b^	24.73 ± 0.25 ^b^	14.70 ± 0.17 ^b^
	Extract	60.37 ± 1.14 ^a^	9.63 ± 0.42 ^c^	27.87 ± 0.70 ^a^
	Cisplatin	55.97 ± 0.38 ^c^	30.07 ± 0.31 ^a^	10.90 ± 0.66 ^c^
HCT116	Control	55.23 ± 0.25 ^a^	32.83 ± 0.38 ^b^	10.53 ± 0.31 ^b^
	Extract	50.93 ± 0.67 ^b^	10.03 ± 0.06 ^c^	37.37 ± 0.67 ^a^
	Cisplatin	48.80 ± 0.26 ^c^	36.80 ± 0.53 ^a^	11.33 ± 0.31 ^b^
HeLa	Control	69.37 ± 0.32 ^a^	17.80 ± 0.35 ^b^	11.13 ± 0.12 ^c^
	Extract	61.97 ± 1.79 ^b^	9.33 ± 0.76 ^c^	26.80 ± 0.53 ^a^
	Cisplatin	62.17 ± 0.29 ^b^	20.30 ± 0.62 ^a^	15.33 ± 0.21 ^b^
MDA-MB-231	Control	58.93 ± 0.31 ^a^	24.83 ± 0.21 ^b^	13.50 ± 0.36 ^b^
	Extract	57.63 ± 0.55 ^b^	10.27 ± 0.12 ^c^	28.90 ± 0.46 ^a^
	Cisplatin	59.00 ± 0.20 ^a^	30.13 ± 0.21 ^a^	8.83 ± 0.06 ^c^
MCF-7	Control	54.03 ± 0.21 ^a^	30.03 ± 0.15 ^a^	14.57 ± 0.12 ^b^
	Extract	53.53 ± 0.55 ^a^	9.30 ± 0.26 ^c^	34.27 ± 0.67 ^a^
	Cisplatin	42.47 ± 0.50 ^b^	21.83 ± 0.38 ^b^	34.30 ± 0.30 ^a^

Data are presented as the mean ± SD from three independent experiments. Cells were treated with the extract or cisplatin for 24 h, and cell cycle distribution was determined by propidium iodide staining followed by flow cytometric analysis. Different superscript letters within the same cell line and cell cycle phase indicate statistically significant differences among treatments according to one-way ANOVA followed by Tukey’s post hoc test (*p* < 0.05).

**Table 8:** Inhibition zone diameters (mm) representing the antimicrobial activity of the extract and standard antibiotics (caspofungin and ampicillin) against selected microbial strains.

	Fungi		Gram-Negative		Gram-Positive	
	*C. albicans*	*C. glabrata*	*E. coli*	*P. aeruginosa*	*S. aureus*	*E. faecalis*
Inhibition zone diameter (mm)						
Extract	11.30 ± 0.25 ^b^	11.10 ± 0.12 ^b^	11.50 ± 0.41 ^b^	-	14.10 ± 0.15 ^b^	-
Caspofungin	29.30 ± 0.52 ^a^	31.00 ± 0.16 ^a^	-	-	-	-
Ampicillin	-	-	21.60 ± 0.42 ^a^	17.60 ± 0.42	23.00 ± 1.17 ^a^	29.00 ± 1.00
Minimum inhibitory concentration (µg/mL)						
Extract	800	800	800	>800	400	800
Caspofungin	0.40	0.40				
Ampicillin	-	-	12.50	400	3.12	1.56

Inhibition zone values are presented as the mean ± SD from three independent experiments. Different superscript letters within the same microorganism indicate statistically significant differences among treatments according to one-way ANOVA followed by Tukey’s post hoc test (*p* < 0.05). MIC values are expressed as µg/mL. -, not tested.

## Data Availability

The data presented in this study are available within the article and its Supplementary Materials. Further information is available from the corresponding author upon reasonable request.

## Supplementary Materials

The following supporting information can be downloaded at: https://www.mdpi.com/article/10.3390/ijms27167385/s1.

## Abbreviations

The following abbreviations are used in this manuscript:

ABTS	2,2′-azino-bis (3-ethylbenzothiazoline-6-sulfonic acid)
ATCC	American Type Culture Collection
CFU	Colony-forming units
DHA	Docosahexaenoic acid
DMEM	Dulbecco’s Modified Eagle Medium
DMSO	Dimethyl sulfoxide
DPPH	2,2-diphenyl-1-picrylhydrazyl
EPA	Eicosapentaenoic acid
ESI	Electrospray ionization
FAMEs	Fatty acid methyl esters
GAE	Gallic acid equivalents
GC-FID	Gas chromatography–flame ionization detection
GC–MS	Gas chromatography–mass spectrometry
LC–MS/MS	Liquid chromatography–tandem mass spectrometry
LOD	Limit of detection
LOQ	Limit of quantification
MIC	Minimum inhibitory concentration
MRM	Multiple reaction monitoring
MTT	3-(4,5-dimethylthiazol-2-yl)-2,5-diphenyltetrazolium bromide
PBS	Phosphate-buffered saline
PI	Propidium iodide
RI	Retention index
RT	Retention time
TE	Trolox equivalents
TPC	Total phenolic content
YPD	Yeast extract peptone dextrose
