# Supplementary material for: Comprehensive Phytochemical Characterization of Echinophora tenuifolia subsp. sibthorpiana Reveals Radical Scavenging, Antimicrobial, and Antiproliferative Activities Associated with Apoptosis and G2/M Cell Cycle Accumulation
Source: Int J Mol Sci. 2026 Aug 18;27(16):7385. doi: 10.3390/ijms27167385 (PMC13513104; doi:10.3390/ijms27167385)
Supplement: Supplementary file 1 [file ijms-27-07385-s001.zip › ijms-4503267-supplementary.pdf]

Comprehensive Phytochemical Characterization of *Echinophora tenuifolia* subsp. *sibthorpiana* Reveals Antioxidant, Antimicrobial, and Antiproliferative Activities Associated with Apoptosis and G2/M Cell Cycle Accumulation

Yılmaz Uğur[1*], Turgay Kolaç[1], Muhammed Dündar[2], Rukiye Zengin[3], Tahir Macit[3], Umay Sena Alan[4], Hüseyin Karcı[2], Zeynep Maraş[4], Feyza Yılmaz Dündar[2], Onural Özhan[5], Selim Erdoğan[4]

[1]Health Services Vocational School, Inonu University, Malatya, 44280, Türkiye
[2]Department of Molecular Biology and Genetics, Inonu University, Faculty of Arts and Sciences, Malatya, 44280, Türkiye
[3]Republic of Türkiye Ministry of Agriculture and Forestry, General Directorate of Agricultural Research and Policies, Apricot Research Institute, Malatya, 44090, Türkiye
[4]Faculty of Pharmacy, Basic Pharmaceutical Sciences Department, Inonu University, Malatya, 44280, Türkiye
[5]Faculty of Medicine, Department of Medical Pharmacology, Inonu University, Malatya, 44280, Türkiye

*Correspondence: yilmaz.ugur@inonu.edu.tr

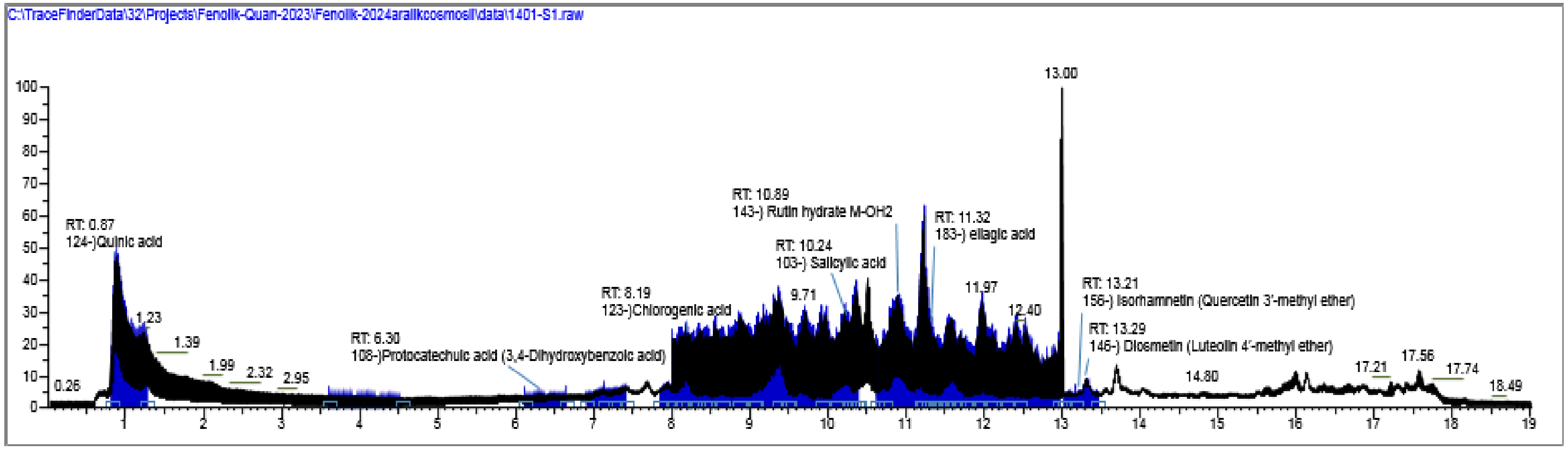

Figure S1. Representative LC–MS/MS chromatogram of the *Echinophora tenuifolia* subsp. *sibthorpiana* extract.

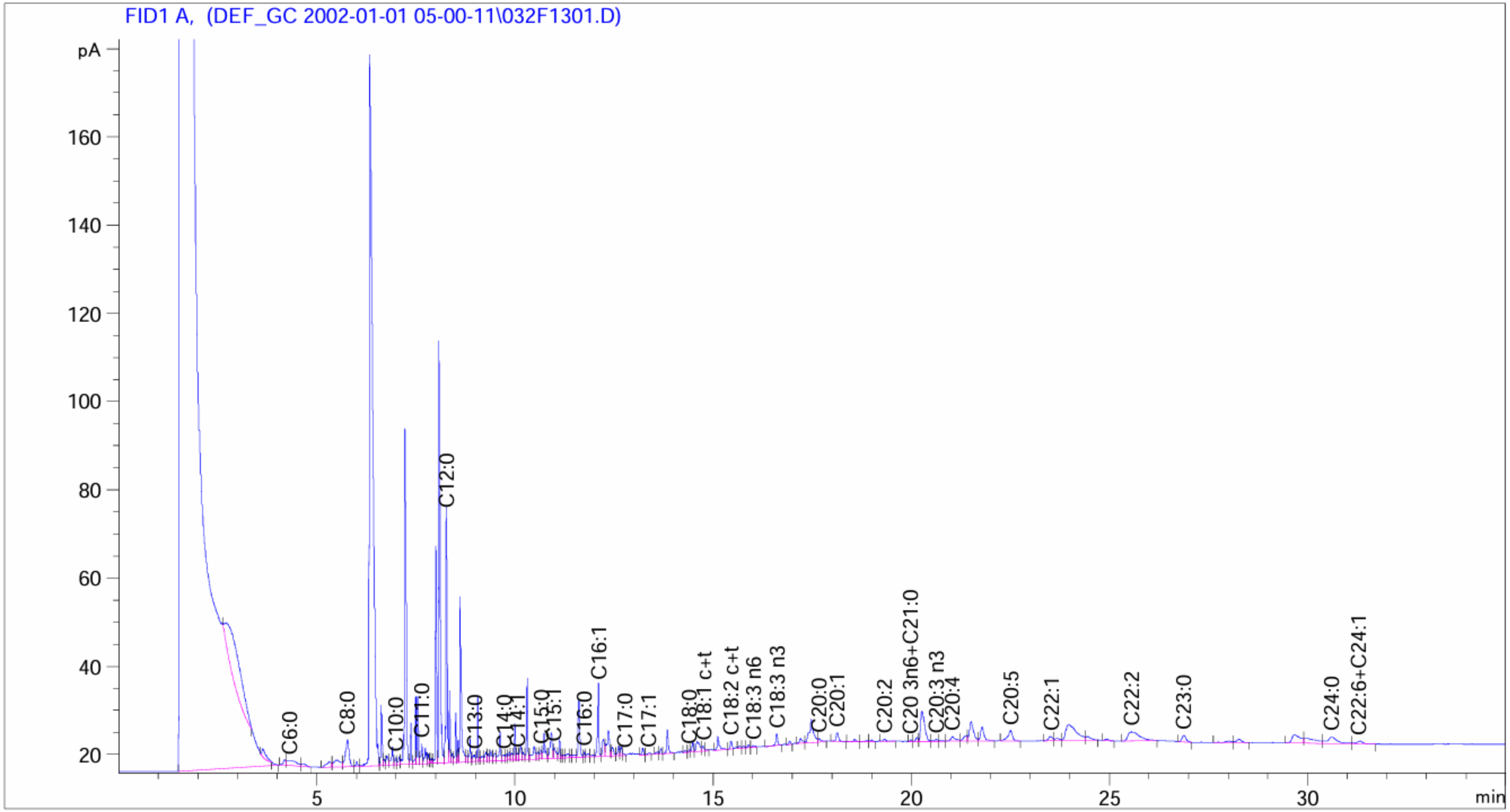

Figure S2. Representative GC–FID chromatogram of the fatty acid composition of *Echinophora tenuifolia* subsp. *sibthorpiana*.

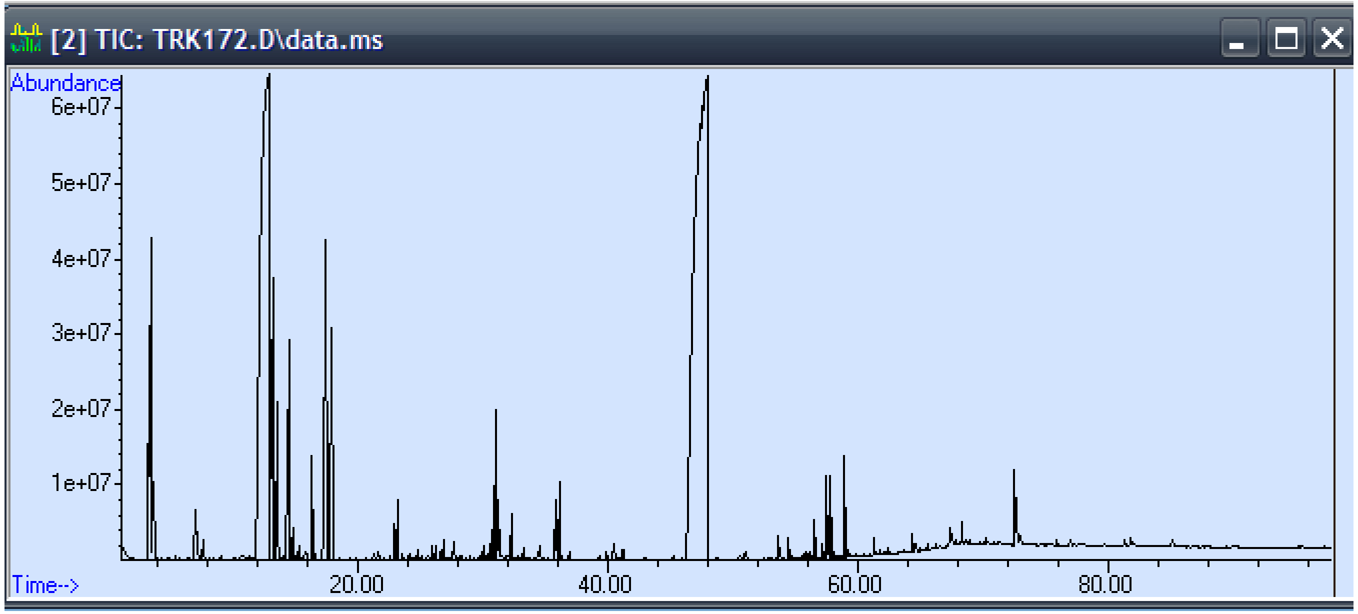

Figure S3. Representative GC–MS chromatogram of the essential oil from *Echinophora tenuifolia* subsp. *sibthorpiana*.

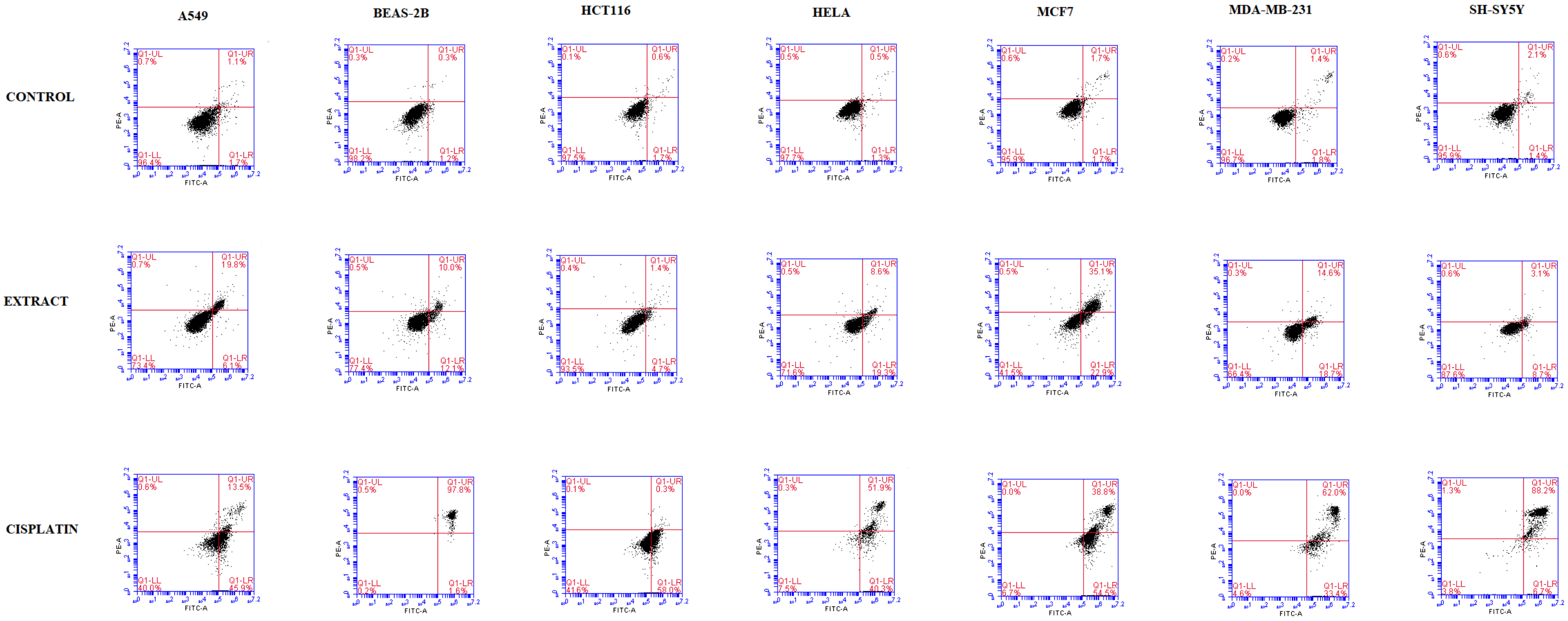

Figure S4. Representative Annexin V–FITC/PI flow cytometric dot plots showing apoptosis induction in cancerous and non-cancerous cell lines following treatment with *Echinophora tenuifolia* subsp. *sibthorpiana* extract. Representative dot plots of Annexin V–FITC/PI staining in A549, BEAS-2B, HCT116, HeLa, MCF-7, MDA-MB-231, and SH-SY5Y cells following 24 h treatment with E. tenuifolia extract or cisplatin. Untreated cells served as controls. Lower left quadrant (Annexin V−/PI−) represents viable cells, lower right quadrant (Annexin V+/PI−) early apoptotic cells, upper right quadrant (Annexin V+/PI+) late apoptotic/secondary necrotic cells, and upper left quadrant (Annexin V−/PI+) necrotic cells. The percentages shown in each quadrant indicate the distribution of the corresponding cell population.

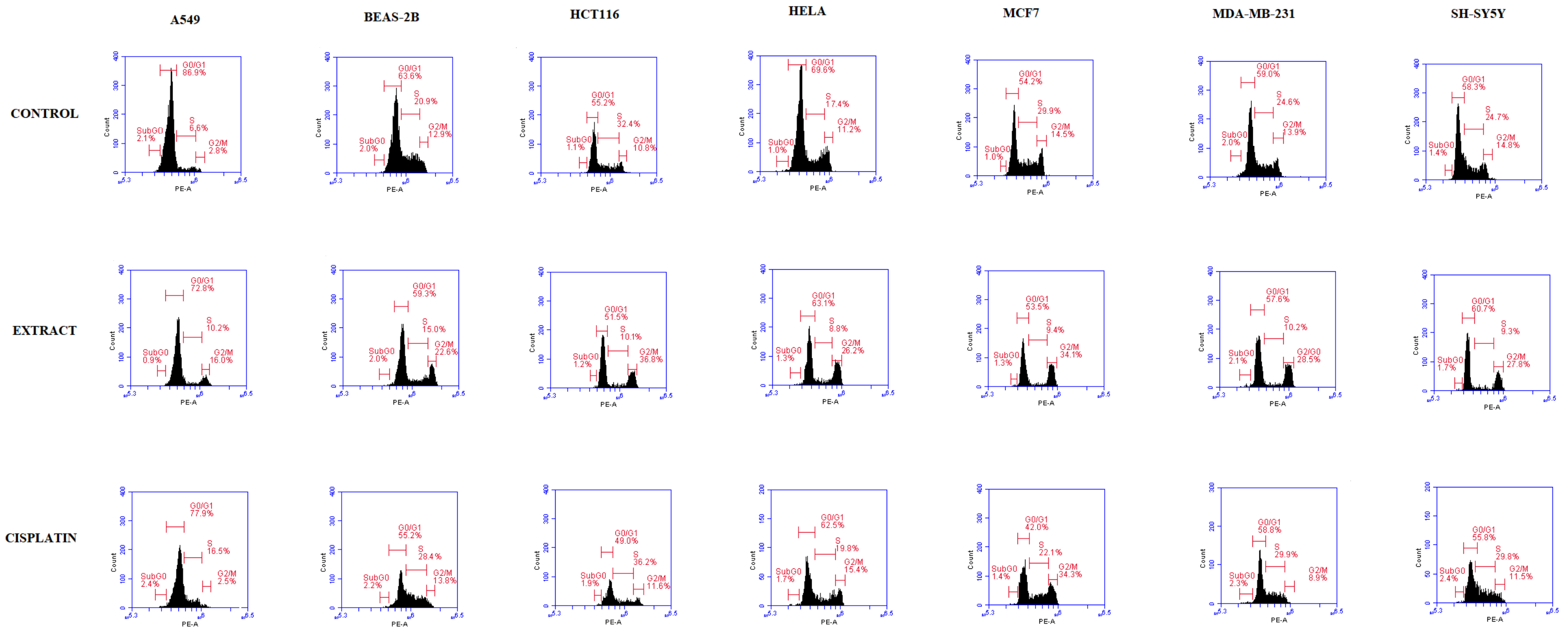

Figure S5. Representative flow cytometric histograms illustrating the effects of *Echinophora tenuifolia* subsp. *sibthorpiana* extract on cell cycle distribution in cancerous and non-cancerous cell lines. Representative DNA-content histograms obtained by propidium iodide staining following 24 h treatment with E. tenuifolia extract or cisplatin in A549, BEAS-2B, HCT116, HeLa, MCF-7, MDA-MB-231, and SH-SY5Y cells. Untreated cells served as controls. Cell populations corresponding to the SubG0, G0/G1, S, and G2/M phases are indicated within each histogram. Percentages shown in the histograms represent the proportion of cells in each phase of the cell cycle.
